# Shea Nut Oil Triterpene Concentrate Attenuates Knee Osteoarthritis Development in Rats: Evidence from Knee Joint Histology

**DOI:** 10.1371/journal.pone.0162022

**Published:** 2016-09-01

**Authors:** Jen-Hsin Kao, Sheng-Hsiung Lin, Chun-Fu Lai, Yu-Chieh Lin, Zwe-Ling Kong, Chih-Shung Wong

**Affiliations:** 1 Department of Medical Research, Cathay General Hospital, Taipei, Taiwan; 2 Graduate Institute of Medical Science, National Defense Medical Center, Taipei, Taiwan; 3 Department of Family and Community Medicine, Tri-Service General Hospital, National Defense Medical Center, Taipei, Taiwan; 4 Department of Family Medicine, Tri-Service General Hospital Songshan Branch, Taipei, Taiwan; 5 Department of Pathology, Tri-Service General Hospital, National Defense Medical Center, Taipei, Taiwan; 6 Department of Pathology, Taoyuan Armed Forces General Hospital, Taoyuan, Taiwan; National Defense Medical Center, Taipei, Taiwan; 7 Department of Food Science, National Taiwan Ocean University, Keelung, Taiwan; 8 Department of Anesthesiology, Cathay General Hospital, Taipei, Taiwan; 9 Department of Pharmacology, National Defense Medical Center, Taipei, Taiwan; Umea Universitet, SWEDEN

## Abstract

**Background:**

Shea nut oil triterpene concentrate is considered to have anti-inflammatory and antioxidant properties. Traditionally, it has been used to treat arthritic conditions in humans. This study aimed to investigate the effect of attenuating osteoarthritis (OA)-induced pain and joint destruction in rats by administering shea nut oil triterpene concentrate (SheaFlex75, which is more than 50% triterpenes).

**Methods:**

An anterior cruciate ligament transaction (ACLT) with medial meniscectomy (MMx) was used to induce OA in male Wistar rats. Different doses of SheaFlex75 (111.6 mg/kg, 223.2 mg/kg, and 446.4 mg/kg) were then intragastrically administered daily for 12 weeks after surgery. Body weight and the width of the knee joint were measured weekly. Additionally, incapacitance tests were performed at weeks 2, 4, 6, 8, 10 and 12 to measure the weight bearing of the hind limbs, and the morphology and histopathology of the medial femoral condyles were examined and were evaluated using the Osteoarthritis Research Society International (OARSI) scoring system.

**Results:**

This study showed that SheaFlex75 reduced the swelling of the knee joint with OA and rectified its weight bearing after ACLT plus MMx surgery in rats. Treatment with SheaFlex75 also decreased ACLT plus MMx surgery-induced knee joint matrix loss and cartilage degeneration.

**Conclusion:**

SheaFlex75 relieves the symptoms of OA and protects cartilage from degeneration. SheaFlex75 thus has the potential to be an ideal nutraceutical supplement for joint protection, particularly for injured knee joints.

## Introduction

Osteoarthritis (OA) is a chronic degenerative joint disorder that can be caused by aging, injury, overuse, obesity, a genetic defect or other conditions. This disorder is characterized by erosion and loss of the articular cartilage, overgrowth of subchondral bone, development of osteophytes, and synovitis [1]. Pain is the main symptom of OA, and patients complain more about pain than about any other symptom. Approximately 50% of symptomatic patients describe pain at rest, and nearly all patients have use-related pain when bearing weight and moving the affected joint [1,2]. OA pain leads to disability and a reduction in the quality of life. However, the recommended treatments, such as acetaminophen, nonsteroidal anti-inflammatory drugs (NSAIDs), and steroids, provide incomplete relief [3]. Therefore, effective pain relief in OA is currently lacking.

Animal models of OA that either occur naturally or are induced surgically or chemically have been used to study the pathogenesis of cartilage degeneration and potential therapeutic modulation of the disease [4]. The pathology and pathogenesis of naturally occurring OA are similar to what occurs in humans with age. However, OA is a slowly progressing disease, and a long time period is needed for drug testing or a pathogenesis study [4]. By contrast, in surgical or chemical OA models, the disease can be rapidly induced, and the manifestations are less variable [5]. An intra-articular injection of iodoacetic acid, an inhibitor of aerobic glycolysis that kills chondrocytes, has also been used to induce OA [6,7]. However, cases induced in this manner are considered to be models for cartilage damage, inflammation and joint pain caused by chemical-induced chondrocyte death, rather than OA models [8]. Meanwhile, the surgically induced instability model of OA mimics the pathogenesis and pathology of OA induced by joint injury in humans because rupture or tear of the anterior cruciate ligament (ACL) is one of the main causes of OA in younger individuals [9]. Surgical methods to induce OA include induction of medial meniscus tear [10–12], ACL tear [13–15], partial medial meniscus tear [16], and ACL tear with partial medial meniscus tear [14,17]. Surgically induced OA in rats results in rapidly progressive degenerative changes in the cartilage, characterized by chondrocytes and proteoglycan loss, fibrillation, osteophytes formation, and chondrocyte cloning [4,18]. Surgically induced OA also produces significant mechanical hyperalgesia and tactile allodynia in rats [12]. In the present study, we used ACL transaction (ACLT) plus medial meniscectomy (MMx) to induced rapidly progressing OA in rats.

*Vitellaria paradoxa* (formerly *Butyrospermum parkii*), commonly known as the shea tree, is a slow-growing tree from equatorial Africa [19]. Its fruit is used as a source of nutrition, and the fat extracted, known as shea butter, is currently used topically in the cosmetics industry. Shea nut oil contains a high level of unsaponifiable material, and the greatest proportion of this material consists of triterpene alcohols [20,21]. Natural triterpenes from plants and shea nut oil are considered to have anti-inflammatory and antioxidant properties [22,23]. SheaFlex75 is produced from shea nut oil through a series of solvent extractions to yield a high concentration of unsaponifiable matter (50–70%). The composition of SheaFlex75 includes α-amyrin, β-amyrin, butyrospermol, lupeol, and other triterpene alcohols [21]. SheaFlex75 has commonly been used to treat arthritic conditions in humans [24,25]. However, the mechanism by which SheaFlex75 induces pain relief is still unknown. In the present study, we investigate the effect of SheaFlex75 on OA-induced pain and joint structural changes in an ACLT plus MMx OA model.

## Materials and Methods

### Osteoarthritis Animal Model

All animal care and experimental protocols complied with institutional and international standards (Principles of Laboratory Animal Care, National Institutes of Health) and were approved by the Institutional Animal Care and Use Committee of Cathay General Hospital (Taiwan, ROC). Adult male Wistar rats (330–350 g, from BioLASCO Taiwan Co., Ltd, Yilan, Taiwan) were kept in an animal room with a 12 h light/dark cycle at a temperature of 25±2°C and 55% humidity. A standard diet and water were provided *ad libitum*. ACLT plus MMx was performed to induce OA. For this purpose, the rats were anesthetized with pentobarbital (65 mg/kg, i.p.), and the hair on the right knee was clipped. An incision was made in the medial aspect of the joint capsule (anterior to the medial collateral ligament), the ACL was transacted, and the medial meniscus was removed. Following surgery, the joint was irrigated with normal saline, the capsule was sutured with 4–0 vicryl, and the skin was closed with 4–0 nylon mattress sutures. In sham-operated rats, an incision was made in the medial aspect of the joint capsule to expose the ACL, but the ACL was not transacted, and the medial meniscus was not removed. The rats were supplied with supplemental heat and were monitored until recovery from anesthesia. The rats were also checked daily regarding their general health and for pain, discomfort and infection in the post-operative period, and cefazolin (20 mg/kg) was injected intraperitoneally before the surgery and twice daily for 3 days after the surgery to prevent infection.

### Experimental Design and SheaFlex75 Administration

**Fig 1:**
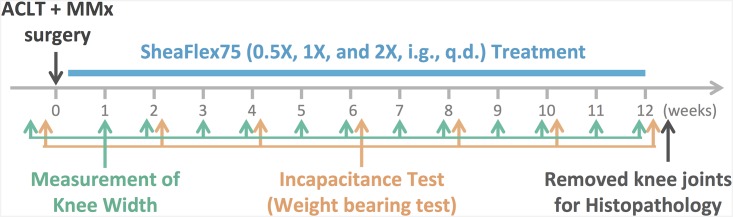
Experimental design and time course of the study.

As shown in Fig 1, the ACLT plus MMx surgery was performed at week 0. The animals were then intragastrically treated with 0.5X SheaFlex75 (111.6 mg/kg), 1X SheaFlex75 (223.2 mg/kg), or 2X SheaFlex75 (446.4 mg/kg) every morning for 12 weeks following the surgery. Their body weight was measured weekly before and after the surgery, and the width of the knee joint was measured using calipers (AA847R, Aesculap, Eindhoven, The Netherlands) before the surgery and every week for 12 weeks after the surgery. Additionally, incapacitance tests were performed before the surgery and at 2, 4, 6, 8, 10 and 12 weeks after the surgery. The animals were sacrificed at week 12, and their knees were dissected after all tests were completed.

### Incapacitance Test (Weight-Bearing Test)

Weight-bearing ability was measured using an incapacitance apparatus (Linton Instrumentation, Norfolk, UK) to detect changes in postural equilibrium. In particular, the rats were stood on their hind paws in a box containing an inclined plane (65° from horizontal) that was placed above the incapacitance apparatus; the weight that the animals applied to each hind limb was measured independently by the apparatus. Three to five measurements were taken for each rat, and the average was calculated after excluding the outlier. The data are expressed as the difference between the weight applied to the limb contralateral to the injury and the weight applied to the ipsilateral limb (Δ Weight).

### Knee Width and Histopathological Examination of Joints

The width of the knee joint was measured using calipers (AA847R, Aesculap, Eindhoven, The Netherlands) every week for 12 weeks after the operation, and the width of the contralateral knee was used as the baseline. At week 12, after all tests were completed, the rats were euthanized with an overdose of pentobarbital, and the knee joints were removed and fixed in 4% paraformaldehyde for 2 days, followed by decalcification in Rapid Decalcifier (Hestion Scientific Pty Ltd., Australia) for 2 weeks. After decalcification, the joints were embedded in paraffin blocks, and serial sagittal histological sections (5 mm) were obtained using a Leica 2065 rotatory microtome (Leica Instruments, Wetzlar, Germany). Hematoxylin/eosin (H&E) staining was then used to examine the morphological changes. The severity of the articular cartilage damage was evaluated using the modified Osteoarthritis Research Society International (OARSI) scoring system [8]. The cartilage matrix loss width, the cartilage degeneration score, the total and significant cartilage degeneration widths, and the zonal depth ratio of the lesions were specifically evaluated [8].

### Statistical Analysis

The data are expressed as the mean ± S.E.M. Two-way ANOVA and Tukey’s multiple comparisons test were used to analyze the data.

## Results

### Neither ACLT plus MMx Surgery nor SheaFlex75 Treatment Altered the Body Weight of the Rats

**Fig 2:**
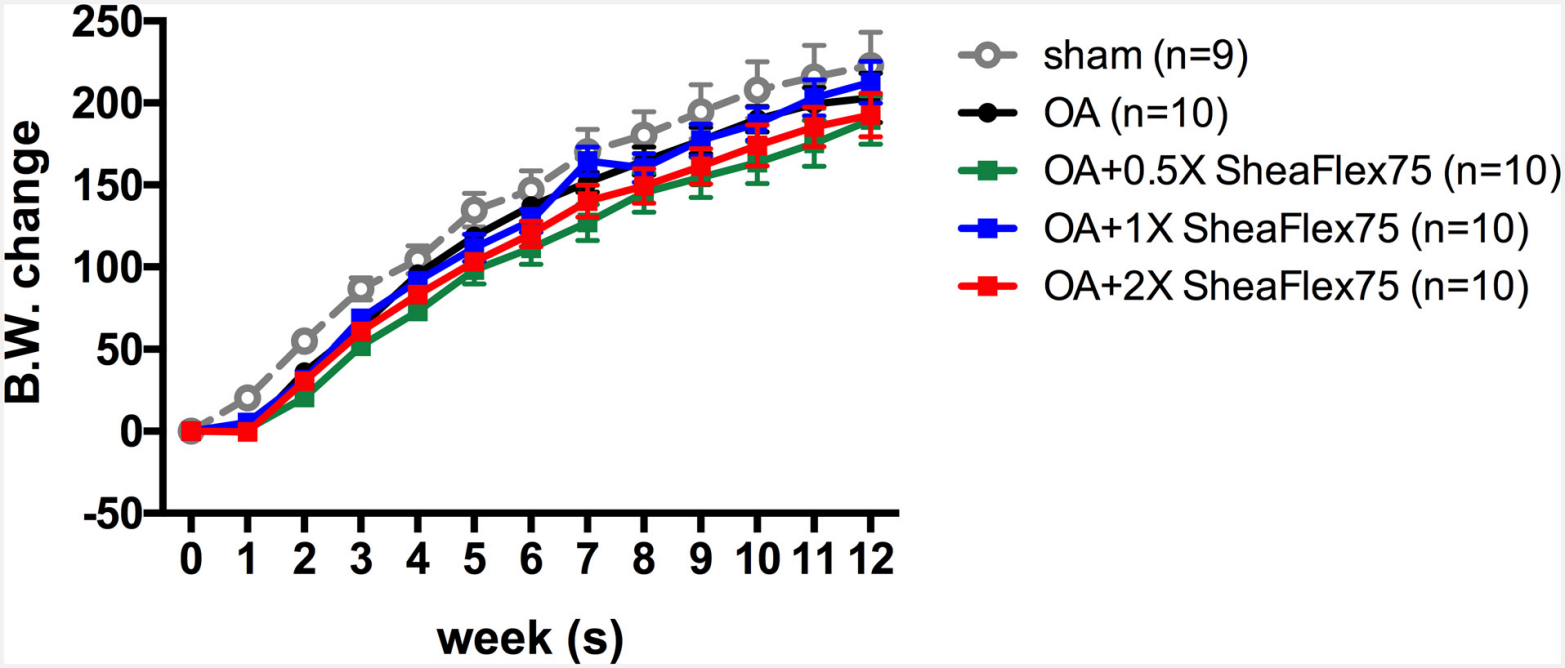
Effect of SheaFlex75 treatment on the body weight of rats that underwent ACLT plus MMx surgery. The data are expressed as the mean ± S.E.M., and two-way ANOVA and Tukey’s multiple comparisons test were used to analyze the data.

No rat showed abnormal behavior, and all survived for 12 weeks until sacrifice. As shown in Fig 2, growth was sustained in all 5 groups. At the end of the experiment, the weights were 222.78±20.23 g in the sham group, 203.20±15.19 g in the OA group, 189.50±14.87 g in the OA + 0.5X SheaFlex75 group, 212.60±12.86 g in the OA + 1X SheaFlex75 group, and 192.50±13.41 g in the OA + 2X SheaFlex75 group. There were no significant differences among the groups, which indicates that neither ACLT plus MMx surgery nor SheaFlex75 treatment altered the body weight of the rats.

### SheaFlex75 Treatment Decreased Hind Paw Weight Distribution in Rats That Underwent ACLT plus MMx

**Fig 3:**
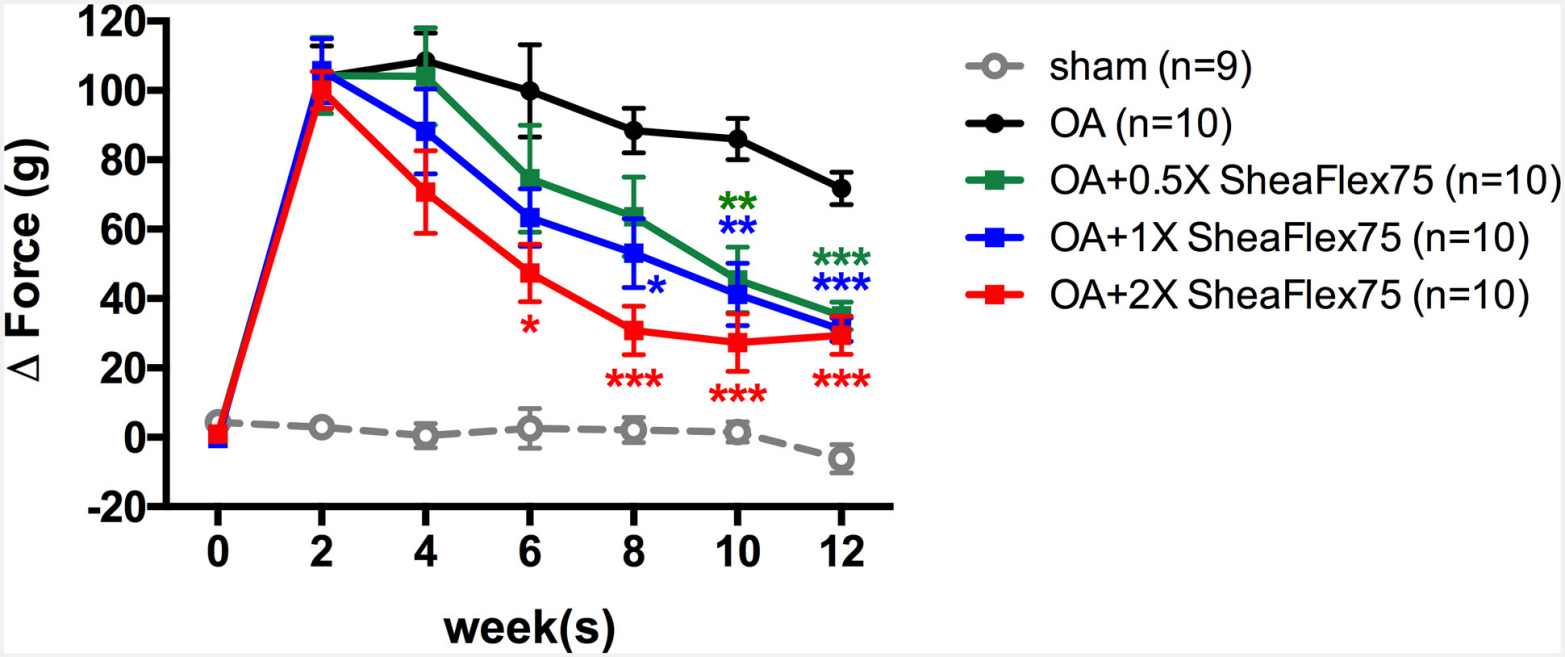
Effect of SheaFlex75 treatment on the weight-bearing distribution of the hind limbs in rats that underwent ACLT plus MMx. An incapacitance test was used to measure the weight bearing of the hind limb before and every 2 weeks after surgery for 12 weeks. The data are the difference between the weights applied to the contralateral and ipsilateral limbs, expressed as the mean ± S.E.M. Two-way ANOVA and Tukey’s multiple comparisons test were used to analyze the data. *p<0.05, **p<0.01, ***p<0.001, when compared to the OA group.

Unilateral ACLT plus MMx surgery induced hind limb weight-bearing alterations in the rats (Fig 3). The difference between the weight placed on the contralateral limb and that placed on the ipsilateral limb significantly increased after ACLT plus MMx surgery in the OA group (from 0.6±1.2 g at week 0 to 103.9±9.0 g at week 2, *p*<0.0001). In contrast, hind limb weight bearing did not change in the sham group (4.4±1.0 g at week 0 and 2.9±2.3 g at week 2). After a daily 2X SheaFlex75 treatment for 6 weeks, the hind paw weight distribution significantly decreased (47.3±8.3 g) when compared to that in the OA group (99.8±13.4 g, *p*<0.0001), and the maximum effect was reached after 8 weeks (30.6±7.0 g, *p*<0.0001). Fig 3 shows that higher doses of SheaFlex75 induced a more rapid effect. The hind paw weight distribution significantly decreased at week 6 (47.3±8.3 g, *p*<0.0001), week 8 (53.1±9.9 g, *p* = 0.0165) and week 10 (45.4±9.4 g, *p* = 0.035) after 2X, 1X, and 0.5X SheaFlex75 treatments, respectively. However, at week 12, there was no difference in weight bearing among the three doses used in this study. Doses of 0.5X, 1X and 2X SheaFlex75 elicited a similar decrease in the hind paw weight distribution in rats that underwent ACLT plus MMx (35.0±4.1 g, 31.0±3.4 g, and 29.4±5.5 g in the 0.5X SheaFlex75, 1X SheaFlex75, and 2X SheaFlex75 groups, respectively).

### SheaFlex75 Treatment Decreased ACLT plus MMx Surgery-Induced Knee Swelling

**Fig 4:**
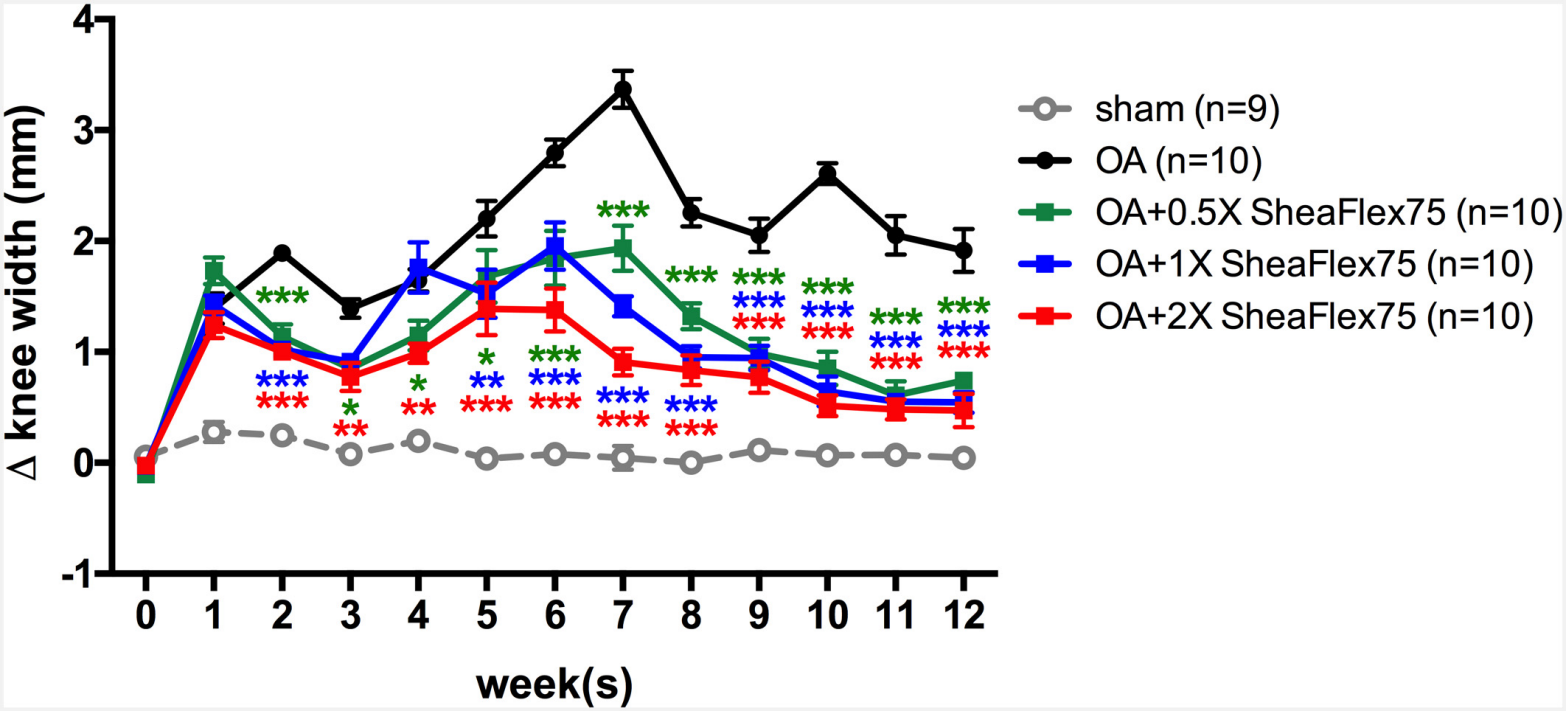
Effect of SheaFlex75 treatment on the knee joint width in rats that underwent ACLT plus MMx. The widths of the bilateral joints were measured before and weekly after surgery. The data are the difference between the widths of the contralateral and ipsilateral knees, expressed as the mean ± S.E.M. Two-way ANOVA and Tukey’s multiple comparisons test were used to analyze the data. *p<0.05, **p<0.01, ***p<0.001, when compared to the OA group.

As shown in Fig 4, the sham operation did not induce knee swelling in the rats. In contrast, the ACLT plus MMx surgery induced significant knee swelling in the OA group (0.06±0.04 mm at week 0; 0.28±0.09 mm at week 1; 0.25±0.05 mm at week 2). The difference in knee width increased from -0.03±0.06 mm before to 1.90±0.06 mm 2 weeks after ACLT plus MMx surgery (*p*<0.0001). The ACLT plus MMx surgery-induced knee swelling lasted for 12 weeks in the OA group (1.92±0.19 mm at week 12, *p* = 0.0002). In contrast, after daily SheaFlex75 treatment for 2 weeks, ACLT plus MMx surgery-induced knee swelling significantly decreased (1.14±0.11 mm, *p* = 0.0003 after 0.5X SheaFlex75 treatment; 1.02±0.07 mm, *p*<0.0001 after 1X SheaFlex75 treatment; and 1.00±0.08 mm, *p*<0.0001 after 2X SheaFlex75 treatment); specifically, 0.5X, 1X, and 2X doses of SheaFlex75 elicited a significant anti-swelling effect in knees with OA after daily treatment.

### SheaFlex75 Treatment Decreased ACLT plus MMx Surgery-Induced Matrix Loss and Cartilage Degeneration

**Fig 5:**
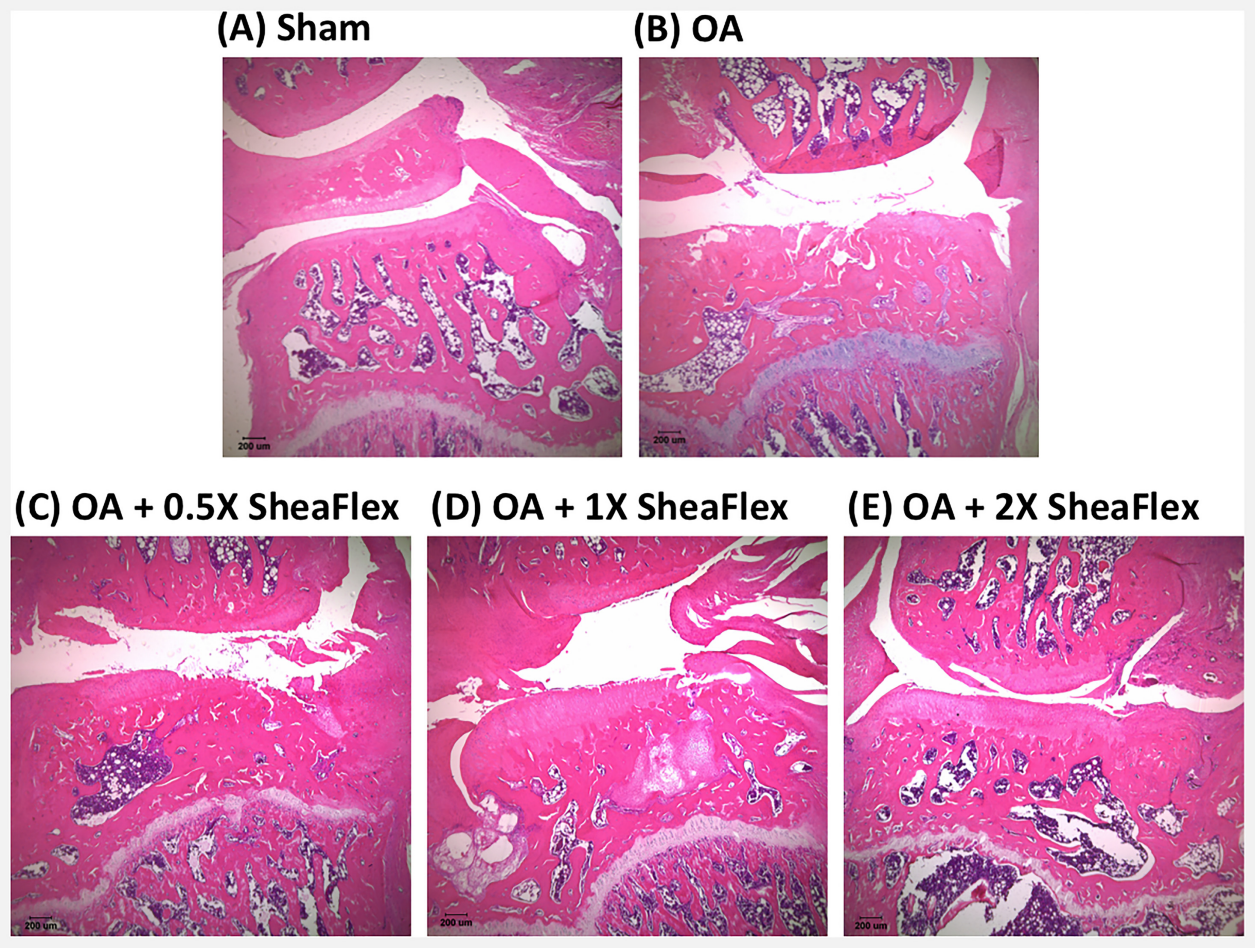
Representative histological sections from the weight-bearing part of the medial tibial plateau from the sham (A), OA (B), OA + 0.5X SheaFlex75 (C), OA + 1X SheaFlex75 (D), and OA + 2X SheaFlex75 (E) groups. The knee joint samples were collected 12 weeks after sham or ACLT plus MMx surgery. The sections were H&E stained.

Fig 5 shows representative H&E-stained sections of cartilage from the right medial condyle of the femur and tibia of the sham, OA, and SheaFlex75-treated groups at week 12. As shown in Fig 5A and Table 1, the surface of the cartilage was smooth in the sham group, and there was no cartilage matrix loss or degeneration. In contrast, the cartilage surface was irregular in the OA group, and significant cartilage loss can be observed (Fig 5B). Table 1 shows that the knee width and the area and depth of cartilage degeneration significantly increased in the OA group compared to the sham group. As shown in Fig 5D and 5E, the surface of the cartilage in the SheaFlex75-treated groups appeared to have fewer macroscopic changes than in the OA group. The cartilage degeneration score, the total and significant cartilage degeneration widths, and the zonal depth ratio of the lesions were also significantly lower after 2X SheaFlex75 treatment than in the untreated group after ACLT plus MMx surgery (Table 1).

**Table 1:** Histopathological changes in the knees of the five studied groups.

	Sham (n = 4)	OA (n = 4)	OA + 0.5X SheaFlex75 (n = 4)	OA + 1X SheaFlex75 (n = 5)	OA + 2X SheaFlex75 (n = 4)
**1. Cartilage matrix loss (mm)**	0**	3.0 ± 0.8	2.7 ± 0.8	1.8 ± 0.2	1.3 ± 0.3
**2. Cartilage degeneration score**	0***	10.2 ± 1.0	5.8 ± 2.3*	3.1 ± 1.0**	3.2 ± 0.5**
**3. Total cartilage degeneration width (mm)**	0***	2.2 ± 0.2	1.7 ± 0.4	1.7 ± 0.2	1.1± 0.2*
**4. Significant cartilage degeneration width (mm)**	0***	1.5 ± 0.16	0.8 ± 0.3*	0.1 ± 0.1***	0.2 ± 0.1***
**5. Zonal depth ratio of lesions**	0**	1.7 ± 0.3	0.8 ± 0.4*	0.5 ± 0.2**	0.4 ± 0.1**

*p<0.05, **p<0.01, ***p<0.001, when compared to the OA group.

## Discussion

In this study, we used ACLT plus MMx surgery to induce OA and investigate the anti-inflammatory and pain-reliving effects of SheaFlex75 in rats. The converted doses of SheaFlex75 were 111.6 mg/kg, 223.2 mg/kg, and 446.4 mg/kg in rats, which are equivalent to 0.5X (18 mg/kg), 1X (36 mg/kg), and 2X (72 mg/kg), respectively, the recommended human dose according to the publication “Estimating the maximum safe starting dose in initial clinical trials for therapeutics in adult healthy volunteers” (https://federalregister.gov/a/05-14456). Our data showed that treatment with SheaFlex75 could relieve OA surgery-induced pain and inhibit OA surgery-induced swelling of the knee joint in rats. The histopathology results also showed that treatment with SheaFlex75 decreased ACLT plus MMx surgery-induced matrix loss and cartilage degeneration.

Knee OA is the most common form of arthritis and the most widespread disease affecting the joint [1]. Symptoms of OA include pain, aching, stiffness, and swelling in or around the joints, and these symptoms cause disability and a reduction in the quality of life [26]. The risk of OA increases with age; however, joint injury can lead to OA in younger individuals [1]. There are no curative therapeutics for OA, and joint function progressively declines until joint replacement is required. The goal of current OA management remains pain relief and improved motor function [3]. Common pharmacological treatments include acetaminophen, oral or topical NSAIDs, intra-articular injections of corticosteroids, and hyaluronic acid for symptom relief [27]. However, NSAIDs increase the risk of upper gastrointestinal and renal toxicity, which limits the long-term use of oral NSAIDs [3]. Several nutraceuticals, such as glucosamine and chondroitin sulfate, have also been used to treat OA [3]. Glucosamine, the substrate in the biosynthesis of proteoglycan, is essential for the reparative processes in cartilage [28]. Thus, glucosamine has been used to relieve OA symptoms for many years [29]. However, recent studies have shown that glucosamine does not reduce joint pain and has no effect on joint space narrowing when compared to placebo in OA patients [29,30]. Meanwhile, chondroitin sulfate, the major component of aggrecan that forms cartilage, has been shown to have anti-inflammatory, anti-apoptosis, and antioxidative effects in vitro [31]. However, there is no evidence showing that chondroitin sulfate alone or combined with glucosamine has a therapeutic effect in humans [30].

Shea nut extract is a nutraceutical that has traditionally been used to treat arthritic conditions in humans. In addition, the United States Food and Drug Administration (FDA) designated refined shea nut oil as generally recognized as safe (GRAS) as a food ingredient in 1998. In the present study, we investigated the pain-relieving effect of SheaFlex75 using an incapacitance test (weight-bearing test), which showed that SheaFlex75 has a significant pain-reliving effect in rats with OA. In fact, the hind paw weight distribution difference decreased by approximately 70% after daily SheaFlex75 intake for 12 weeks. SheaFlex75 intake does not relieve pain immediately, which is the way that analgesics do; however, the weight-bearing test showed that this treatment does improve joint pain in the hind limb in rats with OA after long-term use, indicating that SheaFlex75 has a slow-acting pain reduction effect. Beyond animal models, Chen et al. also found that SheaFlex75 can relieve pain and improve muscle control in the OA-affected knee in human patients [25]. In the present study, we additionally found a reduction in the excitatory amino acid concentration in joint dialysate, which plays an important role in nociceptive transmission, in the OA-affected knee after SheaFlex75 intake for 12 weeks following ACLT plus MMx surgery compared to the untreated knee (preliminary unpublished data).

OA is associated with synovial inflammation, which results in joint edema [32]. Joint width can thus be used to indirectly evaluate the severity of joint inflammation. However, it takes 4–12 weeks to develop cartilage erosion after ACLT plus MMx surgery [8], and the symptoms of OA are not observed until cartilage degeneration. In Fig 4, the knee width increased after surgery and reached its first peak at week 2, which resulted from the inflammation induced by the surgery. After week 2, the knee width decreased slightly due to the recovery from the surgery, and at 4 weeks post-surgery, the knee width increased again, possibly due to the synovial inflammation induced by cartilage loss. According to our present results, SheaFlex75 significantly decreased the knee width after 2 weeks of intake. SheaFlex75 is a patented concentrate comprising approximately 50–70% triterpenes derived from the shea nut; the overall composition mainly includes α-amyrin, β-amyrin, butyrospermol, and lupeol [21]. Triterpenes, which are isolated from the shea nut and other plants, have been shown to possess anti-inflammatory effects [22,33,34]. In particular, lupeol, one of the triterpenes in shea butter and certain plants, has been reported to be an anti-arthritic and anti-inflammatory agent [35]. Lupeol treatment not only decreases inflammation and knee swelling in arthritic animals [36–40] but also improves these animals’ overall condition by providing protection from pain and improving mobility [37,38,40]. Cheras et al. also indicated that the levels of inflammatory marker TNF-α and the cartilage degradation marker CTX-II significantly decreased after SheaFlex75 treatment in OA patients [24]. However, the mechanism underlying the anti-inflammatory effect of SheaFlex75 in rats with OA is still unclear.

Proinflammatory cytokines are known to play an important role in the pathophysiology of OA [32]. Avocado/soybean unsaponifiables (ASUs), natural vegetable extracts made from avocado and soybean oils, are another nutraceutical used in relieving OA symptoms [41]. ASUs decreased NSAID intake and resulted in greater symptom relief than placebo in OA patients [42]. In addition, ASUs have been shown to have anti-inflammatory effects via IL-6, IL-8, prostaglandin E_2_ (PGE2), and metalloproteinase 3 (MMP3) reduction [41,43]. SheaFlex75 is also an unsaponifiable extracted from vegetable oil, and the main ingredient, triterpene, has been shown to have anti-inflammatory effects by suppressing NF-κB activation [23,44]. Based on this information, we hypothesize that anti-inflammatory activity may play a role in the symptom-relieving effect of SheaFlex75. Further studies are needed to explore the underlying mechanisms of SheaFlex75 in OA management, including its side effects after long-term ingestion, in detail.

Articular cartilage breakdown is a major characteristic of late-stage OA. In this study, ACLT plus MMx surgery induced significant matrix loss and cartilage degeneration in rats; in particular, deeper matrix loss and a larger area of cartilage degeneration could be observed in the OA-affected knee 12 weeks after surgery. However, the cartilage degeneration was minor in 0.5X and 1X SheaFlex75-treated rats compared to untreated rats. Furthermore, the rats treated with 2X SheaFlex75 only showed superficial cartilage degeneration. These findings indicate that the SheaFlex75 treatment elicited cartilage-protective effects. However, further studies are needed to better understand the underlying mechanisms of the cartilage-protective effect of SheaFlex75.

Based on the present study, SheaFlex75 ingestion not only relieves the symptoms of OA but also protects cartilage from degeneration. SheaFlex75 has also been assessed and designated as GRAS as a food ingredient by the FDA, so we suggest that SheaFlex75 has the potential to be an ideal nutraceutical for joint protection.
